# Letter from Lydston

**Published:** 1909-04

**Authors:** G. Frank Lydston

**Affiliations:** Chicago


### Correspondence.

### Letter From Lydston.

Chicago, March 30, 1909.

*Editor Texas Medical Journal, Austin, Texas:*

I have been accused by sundry well-meaning persons of undue severity, of levity and of a lack of dignity; aye, even of persecuting a noble, self-sacrificing coul, in my "Medical Frankenstein,"

"Literary Banquo's Ghost," and "The Man in the Glass House." I propose to attempt in my valedictory on the subject of George H. Simmons, which will shortly appear, to rehabilitate myself in the good graces of my critics, by being as gentle, as dignified and as just as the occasion and the subject could possibly warrant. Incidentally, I am going to say a foreword which may not altogether lack interest. In the first place, let me assure those heroes who have for so long been barking at Simmons, and who ran under the house as soon as I fired the first shot at the stronghold of medical politics, that they may now safely come out of hiding. I have assumed the entire burden of responsibility and am resigned to the abuse that I have received and will continue to receive. When the very journals whose battles I have been fighting bravely allude to a "certain man, who," etc., there is no danger that the profession will suspect the yellow dogs under the house of being the "certain," etc. If the editors who have attacked, and those who will attack me, would only have the courage to call me by name, I should indeed be happy. This would stamp me as being of the party opposite to themselves, which would help some. Further, as to undue severity, there are not a few men in the profession who think that I am on the square, and if the editorial heroes would but mention my name in their diatribes, they would help the great cause of liberty a lot. I am willing to meet a square man according to the rules of the game he wishes to play, but I do not usually fight snakes or political autocrats with eight-ounce gloves, Queensberry rules. As for this particular quarry whom I am after, he never fought a fair fight in his life. Should I ever fight as unfairly as he has always done, should I ever be as merciless to those who opposed him as he has always been, should I ever be as unjust to friend and foe who crossed my path as he has always been, then, indeed, would I justly be the target for hostile criticism. I have fought Simmons fairly and squarely, with due notice on all occasions that Gridley was ready to fire. Can you say as much of the battles with which your selfish, cold-blooded career has been checkered, O Simmons? You have fought under the banner of the cross—the "double cross"—have you not?

As to the satire and fun of my various diatribes, I have the illustrious examples of Voltaire, who believed that the way to rid the world of an evil was to satirize and ridicule it out of existence. In such a cause I could not help being satirical, and if the whole situation is not funny, my sense of humor is a minus quality.

The great representative medical association of this country, organized under a code which prohibited quacks, pretenders and irregulars even from membership, is dominated by an apostate homeopath and ex-newspaper advertiser of manifold specialties and of compound oxygen! This man is secretary of the Association, editor of its great journal, dictator of its ethical, political and literary interests and regulator of its therapeutics and materia medica. This man's medical training was obtained early in the 80's at Hahneman Medical College, Chicago. Judging by his subsequent performances and the methods of that school in those days, his medical education must have been very thorough.

"But," you say, "what about those other degrees?" Well, I don't know about all of them, but, knowing as I do how he secured some of them, I can guess the rest—and so can you. Simmons' protagonists have persistently howled:

"Supposing he was a homeopath and a newspaper advertiser? He is now a regular and has professionally reformed."

"Professionally reformed!" "What's bred in the bone," etc. Now, I will admit that George H. Simmons, A. M., M. D., L. M., LL. D., U. S. A., A. M. A., Rex, is officially a "regular" but, I think the profession would like to know how he was "regularized." Ere this is published I will have shown how truly great is Simmons. Obstacles which to some men would be insurmountable are to him light as air. Who but he could have taken a medical course many hundreds of miles away from home without seriously interrupting his practice? This, too, before the days of airships and suburban trolleys! What other man in American medicine ever attained so much learning and so many degrees with so little study? Verily, there be those who were born learned!

I will also admit that the journal of the Association is a brilliant success, and that the Association is growing wealthy, but I do not believe that the argument of the fleshpots will be as strong with those members of the Association who must perforce gaze on your treasury from afar, as it is with the little coterie which basks in the golden rays.

Neither do I believe that the fact that argument that the exposé of Simmons' record has been long delayed and, is, therefore, of no import, will hold water with the rank and file. If Simmons secured his appointments through false pretenses, he is now as guilty as he was when the appointments were made. If he obtained his regular diploma fraudulently (and it was by grace of this diploma that he was appointed), he is as big a fraud today as he was on the day he received his diploma.

Just a word for the benefit of those gentlemen who have accused me of "interested motives." I admit that I despise Simmons and fain would see him sent back to the sage brush, but does this fact justify the halo, rosy wings and coat of whitewash with which certain medical politicians would fain endow him?

As for the insinuations that I have selfish financial interests or personal ambitions to subserve in my attacks on Simmons, they are unconscionable, and the perpetrators well know it. As to the insinuations which have been made to the effect that the proprietary medicine men are paying for my campaign literature, and that I covet Simmons' editorial job, the former is not only a lie, but well known to be a lie by its promulgators, while the latter is too absurd for discussion.

My highest ambition is to purge American medicine of Simmons and his gang of politicians, and I am beginning to have a faint ray of hope. And now let me say a word to the gentlemen who have talked so bravely about my attacks on Simmons: When you accuse me of "skulking," etc., you simply make yourselves ridiculous. Please note that, God willing, I will be at Atlantic City in June, and will have with me for distribution a few copies of my "Jules Verne Outdone," showing how Simmons became a regular. I want to "skulk" some more. Come early and avoid the rush.

G. Frank Lydston.

